# Inhibition of Bacterial Adhesion and Antibiofilm Activities of a Glycolipid Biosurfactant from *Lactobacillus rhamnosus* with Its Physicochemical and Functional Properties

**DOI:** 10.3390/antibiotics10121546

**Published:** 2021-12-17

**Authors:** Mitesh Patel, Arif Jamal Siddiqui, Walid Sabri Hamadou, Malvi Surti, Amir Mahgoub Awadelkareem, Syed Amir Ashraf, Mousa Alreshidi, Mejdi Snoussi, Syed Mohd Danish Rizvi, Fevzi Bardakci, Arshad Jamal, Manojkumar Sachidanandan, Mohd Adnan

**Affiliations:** 1 Bapalal Vaidya Botanical Research Centre, Department of Biosciences, Veer Narmad South Gujarat University, Surat 395007, India; patelmeet15@gmail.com (M.P.); malvisurti92@gmail.com (M.S.); 2 Department of Biology, College of Science, University of Hail, Hail P.O. Box 2440, Saudi Arabia; arifjamal13@gmail.com (A.J.S.); walidsabrimail@gmail.com (W.S.H.); mousa.algladi@gmail.com (M.A.); snmejdi@yahoo.fr (M.S.); fevzi.bardakci@gmail.com (F.B.); arshadjamalus@yahoo.com (A.J.); 3 Department of Clinical Nutrition, College of Applied Medial Sciences, University of Hail, Hail P.O. Box 2440, Saudi Arabia; mahgoubamir22@gmail.com (A.M.A.); amirashrafy2007@gmail.com (S.A.A.); 4 Department of Pharmaceutics, College of Pharmacy, University of Hail, Hail P.O. Box 2440, Saudi Arabia; syedrizvi10@yahoo.com; 5 Department of Oral Radiology, College of Dentistry, University of Hail, Hail P.O. Box 2440, Saudi Arabia; smanojk68@gmail.com

**Keywords:** biosurfactants, *Lactobacillus rhamnosus*, gas chromatography–mass spectrometry, antibiofilm, anti-adhesion, exopolysaccharide, lactic acid bacteria, surface tension

## Abstract

Biosurfactants derived from different microbes are an alternative to chemical surfactants, which have broad applications in food, oil, biodegradation, cosmetic, agriculture, pesticide and medicine/pharmaceutical industries. This is due to their environmentally friendly, biocompatible, biodegradable, effectiveness to work under various environmental conditions and non-toxic nature. Lactic acid bacteria (LAB)-derived glycolipid biosurfactants can play a major role in preventing bacterial attachment, biofilm eradication and related infections in various clinical settings and industries. Hence, it is important to explore and identify the novel molecule/method for the treatment of biofilms of pathogenic bacteria. In the present study, a probiotic *Lactobacillus rhamnosus* *(L. rhamnosus)* strain was isolated from human breast milk. Firstly, its ability to produce biosurfactants, and its physicochemical and functional properties (critical micelle concentration (CMC), reduction in surface tension, emulsification index (% EI24), etc.) were evaluated. Secondly, inhibition of bacterial adhesion and biofilm eradication by cell-bound biosurfactants from *L. rhamnosus* was performed against various biofilm-forming pathogens (*B. subtilis, P. aeruginosa, S. aureus* and *E. coli*). Finally, bacterial cell damage, viability of cells within the biofilm, exopolysaccharide (EPS) production and identification of the structural analogues of the crude biosurfactant via gas chromatography–mass spectrometry (GC–MS) analysis were also evaluated. As a result, *L. rhamnosus* was found to produce 4.32 ± 0.19 g/L biosurfactant that displayed a CMC of 3.0 g/L and reduced the surface tension from 71.12 ± 0.73 mN/m to 41.76 ± 0.60 mN/m. *L. rhamnosus* cell-bound crude biosurfactant was found to be effective against all the tested bacterial pathogens. It displayed potent anti-adhesion and antibiofilm ability by inhibiting the bacterial attachment to surfaces, leading to the disruption of biofilm formation by altering the integrity and viability of bacterial cells within biofilms. Our results also confirm the ability of the *L. rhamnosus* cell-bound-derived biosurfactant to damage the architecture of the biofilm matrix, as a result of the reduced total EPS content. Our findings may be further explored as a green alternative/approach to chemically synthesized toxic antibiofilm agents for controlling bacterial adhesion and biofilm eradication.

## 1. Introduction

In nature, the formation of biofilms on living and non-living materials is an aggregation of surface-related bacterial cells, which are encompassed in an extracellular polymeric substance matrix [1]. Within a biofilm, bacteria display dozens of attributes, which makes them hard to eliminate. In comparison to their planktonic forms, they are phenotypically different mainly in the expression of genes and growth rates [2]. Biofilms portray a protected form of growth, which make bacterial cells 1000-fold more resistant to antibiotics and the immune system of the host than the planktonic form. Consequently, biofilms make bacterial cells stay alive within unfriendly conditions and also to spread and inhabit new environments [3]. Many aspects contribute to this antibiotic resistance, such as changes in physiology, steady growth rate, neutralization of the antimicrobial agents and changes in expression of genes [4]. Other aspects, such as synthesis of extracellular polymers, the age of the biofilm, appearance of small colony variants and dysfunction of the local neutrophils, also play an immense role in the resistance of biofilm bacteria towards the antimicrobial agents [5,6,7]. Moreover, the presence of excessive cell densities within the biofilms significantly enhances the possibility of horizontal gene transfer, which enhances the probability of the appearance of strains with higher resistance or distorted virulence profiles [8].

Biofilms of bacteria are typically pathogenic and responsible for nosocomial infections. About 60 to 80% of chronic infections are due to the formation of biofilm. Presently, biofilm is a serious problem around the globe, which causes a severe impact and ultimately huge losses to the food, dairy, oceanic, aquaculture, beverage, environment and biomedical industries [9,10]. Therefore, biofilm removal is a global challenge that necessitates developing novel natural bioactive compounds to control biofilms, as an alternative to antibiotics or chemically synthesized agents.

Different types of microbes, such as bacteria, fungi, yeast, etc., non-ribosomally synthesize secondary metabolites in their resting and/or active growing stages, which are known as ‘biosurfactants’ [11,12,13]. On the basis of their microbial origin and chemical composition, biosurfactants are mainly classified into five classes, which includes polymeric compounds, neutral lipids, phospholipids, lipopeptides and glycolipids [12,14]. In the present time, biosurfactants are gaining attention from scientists for their application in different fields because of their eco-friendly properties, easy mass production, effectiveness under harsh environmental conditions, selectivity and diversity. Apart from their extensive application in the field of oil recovery, bioremediation and industrial emulsification, nowadays these compounds also display their application in the biomedical field as antimicrobials, anti-adhesives and anticancer agents [15,16,17,18].

Lactic acid bacteria (LAB), commonly known as ‘probiotic bacteria’, are usually presumed to have effective function in keeping good health and immunity in humans. They produce diverse imperative antimicrobial metabolites such as bacteriocins, bacteriocin-like compounds, lactic acid, hydrogen peroxide and biosurfactants, which have an immense number of applications in the biomedical field. Among them, biosurfactants are the important one, which can play a critical role in the inhibition of the adherence ability of numerous pathogens—an essential step for the formation and proliferation of biofilms [19]. Hence, biological compounds with antimicrobial properties and the capability to inhibit the adhesion potential of pathogens on different types of surfaces can be developed as a potent antibiofilm agent. Such biofilms of pathogenic bacteria commonly occur on catheters, silicon-based devices, cardiac devices, surgical wounds and other prostheses [17,20]. Numerous reports have been available on the biosurfactant production ability and application of those biosurfactants in the inhibition of adhesion of microbes, desorption activity and inhibiting the development of biofilm on variety of surfaces, such as silicone, rubber, polypropylene and different biomedical instruments/implants [21,22,23,24,25,26,27,28,29,30].

The biosurfactants derived from different probiotic LAB has extensive applications in these fields. Hence, in vitro assessment of biofilm development prevention or disruption by natural biosurfactants derived from probiotic LAB is a plausible approach that can lead to the discovery of novel antimicrobials. In the present study, probiotic LAB was isolated from human breast milk and a characterization of its functional ability, biosurfactant production and physicochemical properties was carried out. Furthermore, the biomedical potential (antibacterial, antibiofilm and anti-adhesive) of the cell-bound biosurfactant of the isolated LAB was also assessed against various biofilm-forming pathogens.

## 2. Results

### 2.1. Identification and Screening of Promising Biosurfactant-Producing Lactic Acid Bacteria

The probiotic LAB was isolated from a human breast milk sample on MRS agar plates. Based on the morphological and 16S rRNA sequence analysis, the isolated probiotic LAB was identified as *L. rhamnosus.* GenBank sequence database was used after the BLASTn homology run of the obtained nucleotide sequence of the strain MBP002. A more than 99% sequence identity was matched with *L. rhamnosus* against the nucleotide sequence collection in the database. Following successful identification, the nucleotide sequence with accession number MZ496826 was deposited into GenBank database of NCBI.

*L. rhamnosus* is a member of the probiotic *Lactobacillus* genus, which is a short, rod-shape, Gram-positive, homofermentative, facultative anaerobic. Originally, it was considered to be a subspecies of *L. casei*, but genetic research found it to be a separate species in the *L. casei* clade, which also includes *L. paracasei* and *L. zeae* [31,32,33]. Several changes have been made to the taxonomy of the *L. casei* group. In the approved lists of bacterial names [34], *L. casei* was categorized as a single species with five subspecies based on phenotypic features: *L. casei* subsp. *casei*, *L. casei* subsp. *alactosus*, *L. casei* subsp. *pseudoplantarum*, *L. casei* subsp. *tolerans* and *L. casei* subsp. *rhamnosus*. On the basis of DNA–DNA homology, this species was reclassified into three species and two subspecies [35]: (i) *L. casei* (including strains that previously belonged to *L. casei* subsp. *casei*); (ii) *L. paracasei* consisting two subspecies, *L. paracasei* subsp. *paracasei* (including the previous subspecies *L. casei* subsp. *alactosus* and *L. casei* subsp. *pseudoplantarum*) and *L. paracasei* subsp. *tolerans* (including the previous sucspecies *L. casei* subsp. *tolerans*); and (iii) *L. rhamnosus* (subspecies that were previously classified *L. casei* subsp. *rhamnosus*).

The biosurfactant-producing capability of the isolated *L. rhamnosus* cell-free solution was screened via different qualitative and quantitative assays. Firstly, a drop-collapse assay was performed, which is based on the droplet destabilization by surfactants. Accordingly, a drop of a cell-free biosurfactant solution of *L. rhamnosus* was dispensed on oil. On this occasion, if the surfactant is not present in the liquid, the drop stands stable, meaning, from the hydrophobic sites, the polar water molecules are repulsed. Contrastingly, if the surfactant is present in the liquid, the drop will collapse. This is due to the interfacial tension or force between the hydrophobic surface and liquid. In the case of *L. rhamnosus*, the flattened drop of the supernatant placed over the surface of the oil suggested the existence of a biosurfactant. Additionally, an oil-spreading assay was also performed as a confirmatory assay for the validation of the drop-collapse assay result. In this assay, the area of oil displacement is directly proportional to the concentration of surfactants. In the case of *L. rhamnosus*, the oil-spreading assay was conducted in relation to the diameter and time in which the *L. rhamnosus* cell-free biosurfactant solution revealed positive results (Table 1).

### 2.2. Growth Kinetics and Biosurfactant Production

**Figure 1:**
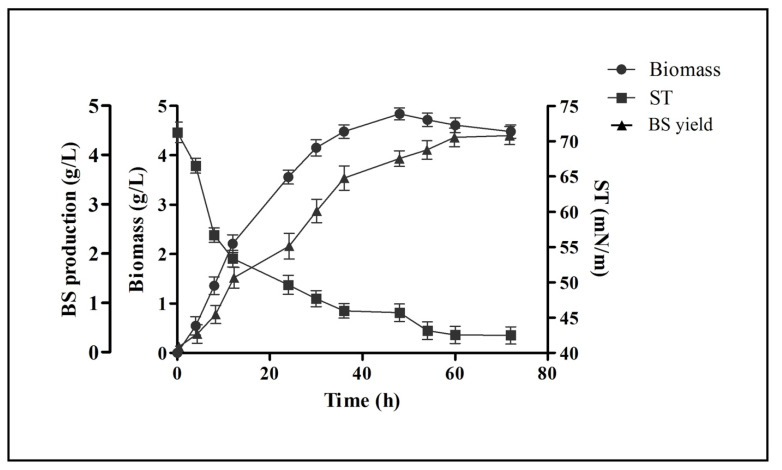
Profiling of the growth kinetics of *L. rhamnosus* in reference to the reduction in surface tension (ST) and production of biosurfactant (BS) at different time intervals. Values are the mean ± SD of three independent experiments.

Biosurfactant production and extraction was carried out in MRS-Lac medium. Figure 1 represents the kinetic profile plot of biosurfactant production by *L. rhamnosus* in this medium. It displayed that the synthesis of biosurfactant was growth-dependent and took place in the log phase. A graph of the surface tension reduction was plotted. Moreover, cell biomass (4.84 ± 0.12 g/L), biosurfactant production (4.32 ± 0.19 g/L) and the highest reduction activity (42.48 ± 1.22 mN/m) was found/noted when the cells entered in their stationary phase. Production of biosurfactant and the reduction in the surface tension were constant up to the termination point of the stationary growth phase.

### 2.3. Physical Properties of the Biosurfactant

**Figure 2:**
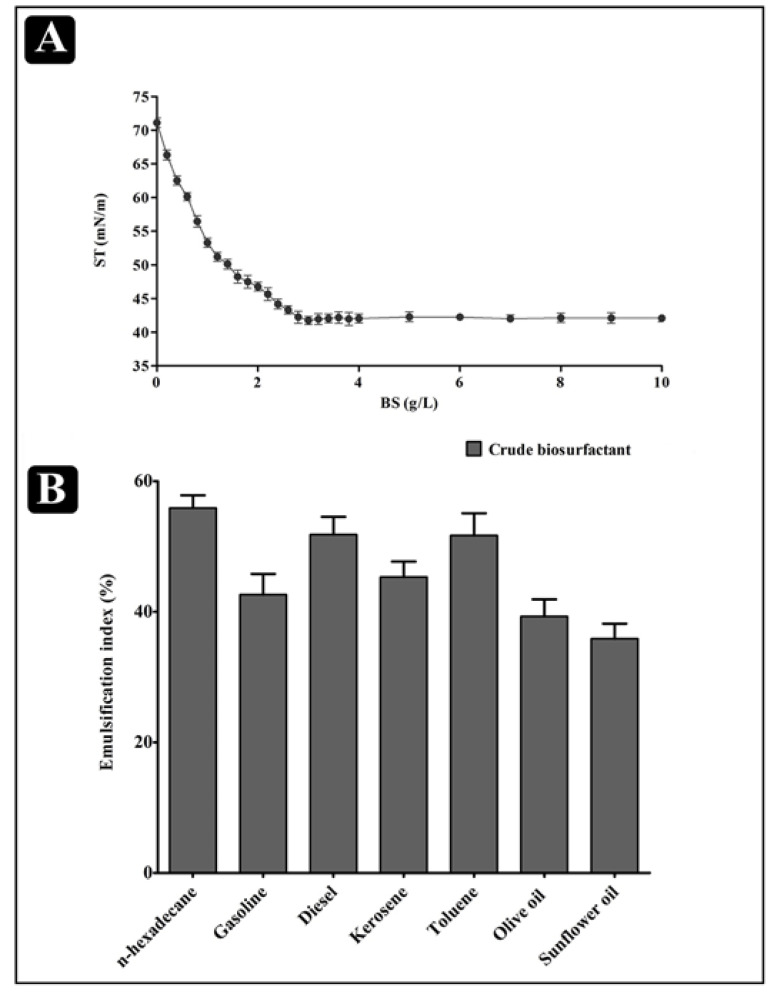
Results of the physical properties of the *L. rhamnosus* crude biosurfactant (BS). (**A**) Progressive decrease in surface tension (ST) with increase in concentration of biosurfactant up to 3.0 g/L. (**B**) %EI24 of the cell-bound biosurfactant against different substrates. Values are the mean ± SD of three independent experiments.

Decreasing the surface tension at the lowest CMC is one of the crucial attributes of an efficient biosurfactant. Extracted cell-bound biosurfactant was further evaluated for reduction in surface tension and CMC value by using a tensiometer (K11, Krus, Germany). A sudden break in the surface tension plot versus the plot of the biosurfactant concentration is defined as the CMC. The biosurfactant produced by *L. rhamnosus* reduced the surface tension from 71.12 ± 0.73 mN/m to 41.76 ± 0.60 mN/m at a CMC of 3.0 mg/mL (Figure 2A). Moreover, the biosurfactant produced by *L. rhamnosus* was also able to emulsify different hydrocarbon substrates, such as n-hexadecane, gasoline, diesel, kerosene, toluene, olive oil and sunflower oil. The highest %EI24 was obtained for an n-hexadecane/biosurfactant emulsion (55.89 ± 1.12), while the lowest %EI24 was obtained for a sunflower oil/biosurfactant emulsion (35.65 ± 1.31) (Figure 2B).

### 2.4. Antibacterial Activity of the L. rhamnosus Crude Biosurfactant

**Figure 3:**
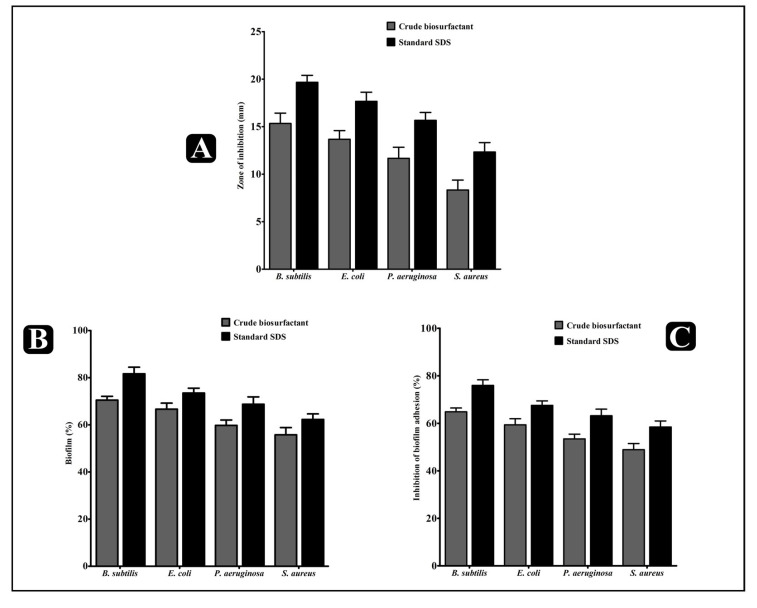
Results of the antibacterial, antibiofilm and anti-adhesion activity of the *L. rhamnosus* crude biosurfactant and standard SDS. (**A**) Antibacterial activity of the *L. rhamnosus* crude biosurfactant (100 mg/mL) and standard SDS surfactant (1% *v*/*v*) against different bacterial pathogens. (**B**) Effect of the *L. rhamnosus* crude biosurfactant and standard SDS surfactant on established biofilms of different bacterial pathogens at their respective MICs. (**C**) Effect of the *L. rhamnosus* crude biosurfactant and standard SDS surfactant on the adherence ability of different bacterial pathogens at their respective MICs. Values are the mean ± SD of three independent experiments.

The inhibitory potential of the crude biosurfactant of *L. rhamnosus* and standard SDS (sodium dodecyl sulfate) was determined via the agar cup/well diffusion method against four different biofilm-forming human pathogens: Gram-negative *E. coli* and *P. aeruginosa*, and Gram-positive *B. subtilis* and *S. aureus*. Both the *L. rhamnosus* crude biosurfactant and standard SDS displayed considerable antibacterial effect against all the tested bacterial pathogens, represented in the form of the zone of inhibition (Figure 3A). The antibacterial potency of the *L. rhamnosus* crude biosurfactant and standard SDS was further evaluated by assessing the minimum inhibitory concentration (MIC) and minimum bactericidal concentration (MBC) against the test pathogens. The values of the *L. rhamnosus* crude biosurfactant MIC were ranged from 12.5 to 50 mg/mL and the MBC values were found two-times higher than the MIC values (Table 2). In turn, the values of the standard SDS MIC ranged from 0.2 to 0.8% and the MBC values from 0.4 to 1.0% (Table 2).

### 2.5. Antibiofilm Potential of the L. rhamnosus Crude Biosurfactant

The antibiofilm potential of the *L. rhamnosus* crude biosurfactant and standard SDS was determined by its capability to impair the preformed biofilms of the test strains and inhibiting their adhesion ability to the surface. Our results showed that the *L. rhamnosus* crude biosurfactant and standard SDS efficiently disrupted the preformed biofilms with an ability to inhibit the adhesion potential of all test strains at MIC. At this concentration, the eradication of the preformed biofilms by the *L. rhamnosus* crude biosurfactant was about 70.49 ± 0.92% for *B. subtilis*, 66.65 ± 1.47% for *E. coli,* 59.78 ± 1.30% for *P. aeruginosa* and 55.77 ± 1.76% for *S. aureus* (vs. 81.65 ± 1.61% for *B. subtilis*, 73.49 ± 1.19% for *E. coli,* 68.72 ± 1.80% for *P. aeruginosa* and 62.28 ± 1.36 % for *S. aureus* for standard SDS). The adhesion potential of the biofilms was also found to decrease with percentage of eradication, being 64.83 ± 1.12% for *B. subtilis*, 59.23 ± 1.47% for *E. coli*, 53.44 ± 1.15% for *P. aeruginosa* and 48.88 ± 1.42% for *S. aureus* (vs. 75.98 ± 1.34% for *B. subtilis*, 67.53 ± 1.08% for *E. coli,* 63.17 ± 1.60% for *P. aeruginosa* and 58.43 ± 1.47% for *S. aureus* for standard SDS) (Figure 3B,C).

### 2.6. Effect of L. rhamnosus Crude Biosurfactant on Bacterial Cells Entrapped in Biofilms

**Figure 4:**
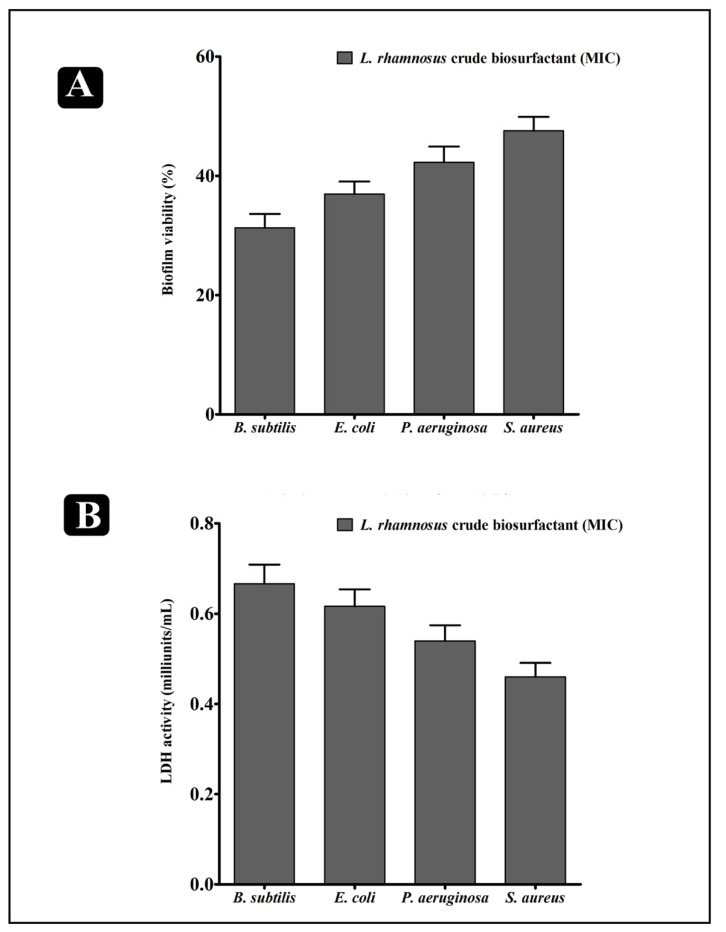
Results of XTT and LDH assays for confirming the antibiofilm potential of the biosurfactant (**A**). Percentage of bacterial viability within biofilms determined by XTT assay at the respective MICs. (**B**) Bacterial cell damage within the biofilm, based on LDH activity upon the treatment of the *L. rhamnosus* crude biosurfactant at the respective MICs. Values are the mean ± SD of three independent experiments.

To explore the prospect that the *L. rhamnosus* crude biosurfactant could decrease the viability of bacteria within biofilms, an XTT (2,3-Bis(2 methoxy-4-nitro-5-sulfophenyl)-5-[(phenyl-amino)carbonyl]-2H-tetrazoliumhydroxide) reduction assay and LDH (lactate dehydrogenase) activity was performed. The obtained results revealed that the viability of all bacteria inside the biofilms were remarkably reduced upon treatment of the *L. rhamnosus* crude biosurfactant, with different susceptivity (Figure 4A). Similarly, LDH activity was also assessed. LDH is the bacterial intrinsic intracellular enzyme that carries out the conversion of lactate into pyruvate and reverse. LDH activity is well-detected when the bacterial cell membrane is not intact. Our results indicated that, upon the treatment of the *L. rhamnosus* crude biosurfactant at the MIC level, LDH activity was found to be elevated in the supernatant of all the test strains. Higher LDH activity was found in *B. subtilis,* whereas lower activity was found in *S. aureus* (Figure 4B). Hence, such results evidently indicates that the *L. rhamnosus* biosurfactant could impair the bacterial cell membrane within the biofilms, eventually leading to bacterial death.

### 2.7. Effect of the L. rhamnosus Crude Biosurfactant on Exopolysaccharide (EPS) Production

**Figure 5:**
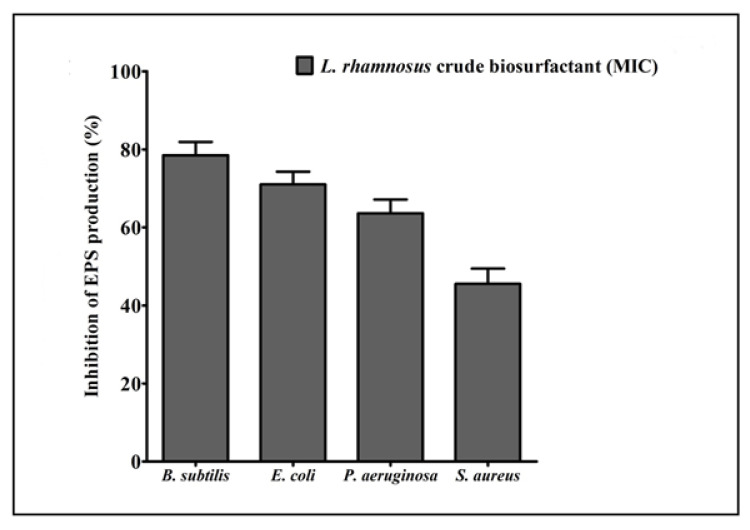
Inhibition of the total EPS production by the test pathogens in the presence of the *L. rhamnosus* crude biosurfactant at their respective MICs. Values are the mean ± SD of three independent experiments.

EPS are biopolymers of bacterial origin and immersed within the biofilm. Biopolymers of EPS develops a matrix and are hydrated by retaining the water and keeps up the cells together within the biofilm. Our results displayed that the production of total EPS was considerably decreased in all test strains after treatment with the *L. rhamnosus* crude biosurfactant (Figure 5).

### 2.8. Microscopic Analysis for the Visualization of the Disrupted Biofilms by Light (LM) and Scanning Electron (SEM) Microscopy

**Figure 6:**
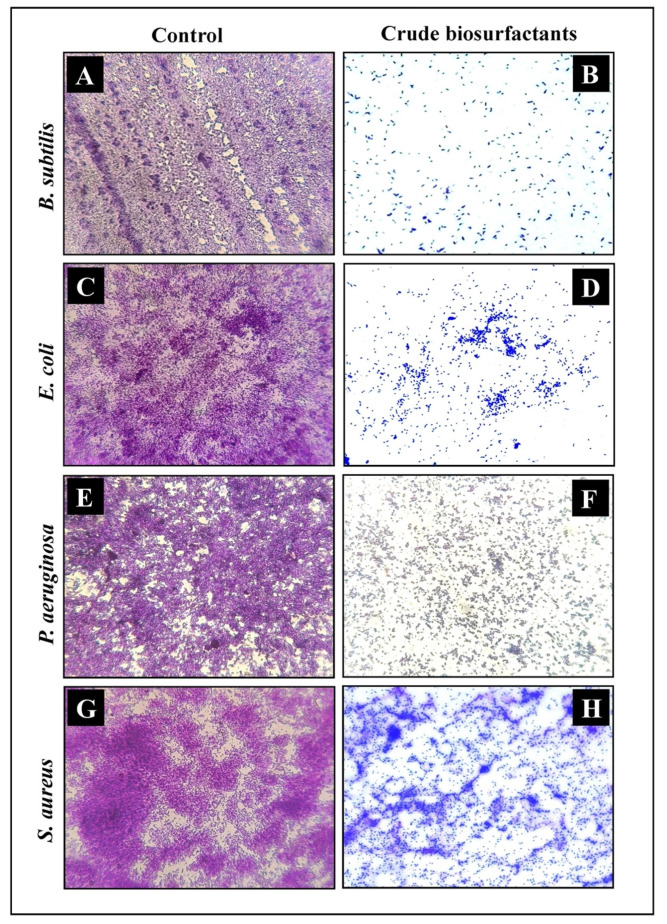
Micrographs of the disrupted matured biofilms of the test pathogens by the *L. rhamnosus* crude biosurfactant formed on a glass surface at their respective MICs under LM. Growth control (**A**,**C**,**E**,**G**); with the *L. rhamnosus* crude biosurfactant (**B**,**D**,**F**,**H**).

**Figure 7:**
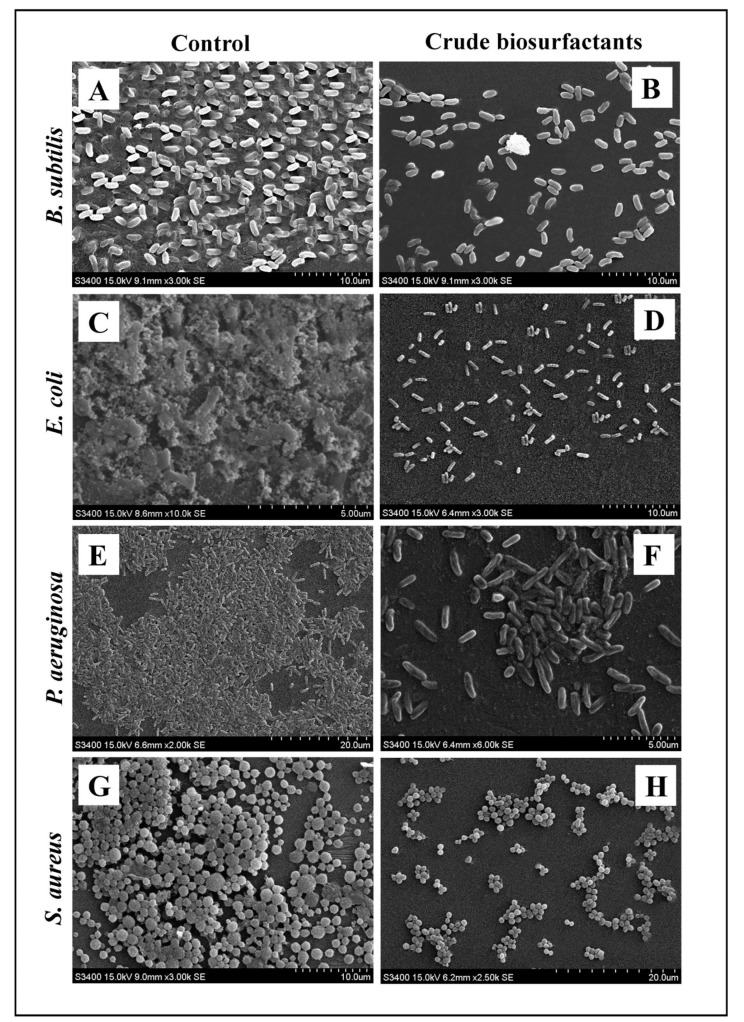
Micrographs of the disrupted matured biofilms of the test pathogens by the *L. rhamnosus* crude biosurfactant formed on a glass surface at their respective MICs under SEM. Growth control (**A**,**C**,**E**,**G**); with the *L. rhamnosus* crude biosurfactant (**B**,**D**,**F**,**H**).

The efficiency and level of biofilm disruption of the test strains by the *L. rhamnosus* crude biosurfactant at its MIC was investigated under LM and SEM. Under light microscopy, the control sample displayed a heavy-knit-like mat of biofilms, while in the presence of the *L. rhamnosus* crude biosurfactant, deterioration in biofilm thickness with lower appearance of micro colonies was observed (Figure 6). Similarly, the biofilm anatomy and surface morphology were confirmed by SEM analysis in the presence and absence of the *L. rhamnosus* crude biosurfactant. In the control group of samples, multi-tiered biofilm growth was seen, while the treatment group with the *L. rhamnosus* crude biosurfactant displayed a reduction in thick aggregation of the tested bacterial cells (Figure 7). This might be due to the impairment of the EPS layer present in the biofilms. These results were further confirmed by the performed EPS assay. Our results of the EPS assay displayed a remarkable reduction in the EPS production of all the test strains treated with the *L. rhamnosus* crude biosurfactant. Altogether, our results have evidently demonstrated the effectiveness of the *L. rhamnosus* crude biosurfactant as a potential, natural and green antibiofilm agent.

### 2.9. Gas Chromatography–Mass Spectrometry (GC–MS) Analysis

**Figure 8:**
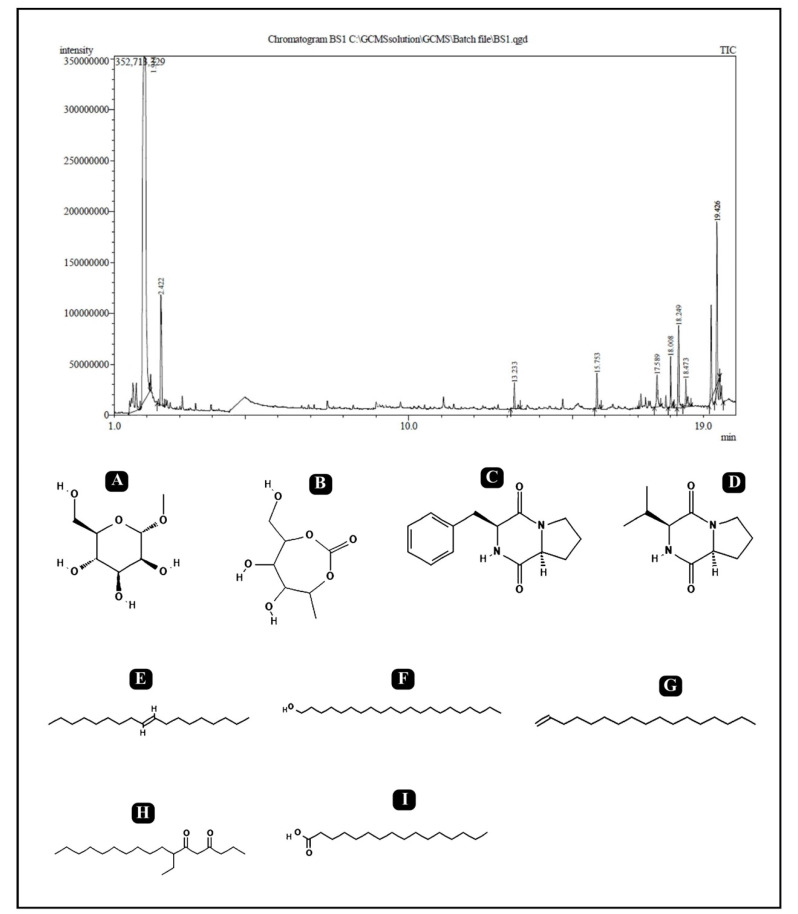
GC–MS analysis of the crude biosurfactants derived from *L. rhamnosus.* Identified compounds: (**A**) Isopropyl alpha-D-mannopyranoside; (**B**) 2,5-Monomethylene-l-rhamnitol; (**C**) (Cyclo(Phe-Pro)); (**D**) Cyclo(L-prolyl-L-valine); (**E**) 9-Octadecene; (**F**) 1-Heneicosanol; (**G**) 1-Heptadecene; (**H**) 7-Ethyl-4,6-heptadecandione; (**I**) *n*-Hexadecanoic acid.

The analytical technique GC–MS consists of gas chromatography combined with mass spectroscopy for the detection of various compounds present in the sample. The GC–MS chromatogram of the *L. rhamnosus* crude biosurfactant shows different peaks, indicating the presence of different compounds (Figure 8). Major peak compounds at the respective retention time were identified from the standard library compound, and are shown in Table 3.

## 3. Discussion

Biofilm can be developed on any kind of biotic and abiotic materials. In liquid medium, it floats on the surfaces and can be in a submerged state. The majority of human diseases are mainly caused by pathogenic bacteria such as *E. coli*, *S. aureus*, *S. epidermis*, *P. aeruginosa*, etc., which are biofilm associated. The diseases caused by/related to biofilm increases the patient morbidity and rate of mortality, leading to a significant economic burden. Within biofilm, bacteria adopt numerous resistant characteristics, which makes them difficult to remove. Moreover, due to the formation of biofilm, the effectiveness of antimicrobial agents is reduced, and the majority of the pathogenic bacteria are no longer susceptible to the available therapeutic agents and antibiotics [1]. Therefore, biofilm formation is a major global concern in current times, which demands novel strategies/alternatives/approaches to control or inhibit biofilm formation.

For infants, the breast milk of humans is recognized as a prime food as it contains all the required nutritional content. Additionally, it provides immunity and gives out to several degrees of protection against infectious diseases [36]. This is due to the presence of diverse types of bioactive molecules (antimicrobial compounds, immune cells and immunoglobulins) that are produced by the microbiota present within the breast milk [37,38]. Several bacterial species, such as *Bifidobacteria*, *Enterococci*, *Lactobacilli*, *Lactococci*, *Micrococci*, *Staphylococci* and *Streptococci*, are reported from human breast milk [39]. Although, there is limited knowledge about the probiotic bacteria and their constituents involved in maintaining the health of the lactating mother and newborn baby. In the present study, the probiotic lactic acid bacterial strain *L. rhamnosus* was isolated from human breast milk, screened, and the functional and biomedical potential of the biosurfactant synthesized by it was characterized.

Biosurfactants have broad applications in food, oil, biodegradation, cosmetic, agriculture, pesticide and medicine/pharmaceutical industries, because of their special properties such as environmentally friendly nature, high selectivity and precise mode of action under harsh environment conditions, such as temperature, pH and salinity [40,41]. Biosurfactants are also reported for their potent antibacterial activity against certain pathogenic bacteria and their biofilms [42]. Biosurfactants cause the formation of pores and ion channels in lipid bilayer membranes, which disrupts the integrity and porosity of the membranes. This leads to membrane disruption and cell death. As a result of this mechanism of action, biosurfactants are active in a range of biological activities, including antibacterial, antifungal, antiviral and antimycoplasma [43,44]. Their biological activity is determined by the structures of these molecules. A lipopeptide may form micellular aggregates or pore channels in the lipid membrane, causing membrane disruption, increased membrane permeability, increased metabolites leakage, membrane structure change, protein conformation change, altering membrane functions and cell death. The dimerization of surfactin into the membrane bilayer causes membrane leakage and instability. Rhamonolipids reduce the lipopolysaccharide content in membranes, increase cell hydrophobicity, alter membrane proteins and disturb surface morphology. Additionally, biosurfactants aid in the detachment of microbial cells from surfaces through sloughing, erosion and abrasion [45]. Furthermore, biosurfactants regulate the quorum sensing signaling (intercellular and intracellular communication) and quorum sensing-dependent activities, such as biofilm formation, motility and pathogenicity, are influenced by this saignaling. Upon binding with ATPase on the mitochondrial membrane, biosurfactants cause apoptosis in several microbial cells cells at low concentrations [46,47]. In the literature, few strains of LAB are reported for their biosurfactant production ability and antimicrobial potential and inhibition of biofilm formation of many pathogenic microorganisms [27,48,49,50,51]. The biosurfactants derived from different probiotic LAB has extensive applications in these fields. Therefore, assessment of biofilm development prevention or disruption by biosurfactants derived from probiotic *L. rhamnosus* might be a plausible approach that can lead to the discovery of novel antimicrobials.

The biosurfactant production ability of *L. rhamnosus* was tested via different qualitative and quantitative methods, such as drop collapse, oil displacement, C-TAB agar plate and emulsification. All these methods are easy to perform and effective to confirm the production of distinct types of biosurfactants from bacteria [52]. The biological surfactants produced by different microbes have advantages over the chemical surfactants, such as biodegradability, low toxicity and environment suitability, which becomes greatly advantageous in different applications. The biosurfactant production from *L. rhamnosus* was started during the log phase and found to be growth dependent. In the stationary phase, the maximum reduction in surface tension (42.48 ± 1.22 mN/m), cell biomass (4.84 ± 0.12 g/L) and production of biosurfactant (4.32 ± 0.19 g/L) was found. The production of biosurfactant and reduction in the value of surface tension were found to be constant till the end of the stationary growth phase (Figure 1). The same kind of pattern in the reduction of surface tension is reported in other studies from LAB strains. Rodrigues et al. (2004) reported the reduction of surface tension from 72 to 39 mN/m and 72 to 37 mN/m, while working with *Lactobacillus fermentum* RC-14 and *Streptococcus thermophilus* A, respectively [28].

One more important parameter of a biosurfactant is the formation of micelles, which is known as the aggregation of amphipathic molecules [16,53,54], and which is necessary to classify an efficient and effective biosurfactant for use and application. The effectiveness is determined from the lowest value at which a reduction in surface tension occurs, whereas CMC is analyzed for the determination of efficiency [55]. Once the concentration of the surfactant is increased in the medium, a reduction in surface tension is started and formation of micelles occurred. The CMC value of the biosurfactant produced by *L. rhamnosus* was found to be 3.0 g/L. The commonly used chemical surfactant SDS has a CMC value of 1.8 g/L, which reduces the surface tension from 72.0 to 37 mN/m [48,56,57]. The CMC value of the biosurfactant produced by *Lactobacillus delbrueckii* in peanut oil cake was around 2 g/L [22]. The effectiveness of any kind of surfactant was determined via its capacity to reduce the surface and interfacial tension of the production medium. Hence, the biosurfactant derived from *L. rhamnosus* is efficient, which can lower the surface tension from 71.12 ± 0.73 mN/m to 41.76 ± 0.60 mN/m. Thus, the results of the present study are in accordance with the other studies in which biosurfactants are extracted from other LAB.

Biosurfactants can alter the necessary functions of the bacterial cell membrane, which are required for the pathogenicity by causing a disruption in the cytoplasmic membranes, which sequentially leads to cell lysis, leakage of important metabolites and disturbs the protein confirmation [58,59]. In the present study, the crude biosurfactant of *L. rhamnosus* was found effective against both Gram-positive and Gram-negative bacteria at different levels. The MIC and MBC values are admirable and relatively economical methods to simultaneously determine the efficacy of the different antimicrobial compounds. The MIC value represent the minimum concentration of the antimicrobial compound that significantly inhibits the growth of bacteria, whereas MBC represent the minimum concentration of any antimicrobial compound that carried out the death of the microbial cell. Generally, antibacterial compounds are recognized as bactericidal, if their MBC values are not more than four times the MIC [60]. According to the obtained MIC values, *B. subtilis, E. coli, P. aeruginosa* and *S. aureus* were susceptible to the crude biosurfactant of *L. rhamnosus*. The values of MIC and MBC was about 12.5 and 25 mg/mL for *B. subtilis* and *E. coli*, 25 and 50 mg/mL for *P. aeruginosa*, and 50 and 1000 mg/mL for *S. aureus*. The obtained results displayed the bactericidal potential of the crude biosurfactant of *L. rhamnosus*.

Apart from antibacterial potential, the *L. rhamnosus*-derived biosurfactant also showed significant results in inhibiting the biofilms of the test pathogens at their respective MICs in a concentration-dependent manner. The *L. rhamnosus*-derived biosurfactant was found to be effectively hampering the adhesion ability, as well as impeding the preformed biofilms of the test strains (Figure 3B,C). The results of the XTT assay also verified that the bacterial cells within the biofilms was inhibited by the crude biosurfactant of *L. rhamnosus* (Figure 4A). The obtained results provided the evidence that the *L. rhamnosus*-derived biosurfactant could also affect the integrity of bacterial cells inside the biofilm. Moreover, the crude biosurfactant also impaired bacterial cells, possibly releasing the intrinsic intracellular enzyme LDH (Figure 4B).

The biomass of biofilm of the pathogenic bacteria was determined via the standard crystal violet method. The results of the present study displayed that the *L. rhamnosus*-derived biosurfactant was efficient in inhibiting the biofilms (Figure 5). This result was further verified by visualizing the biofilms under SEM (Figure 6). Disrupted integrity of the cell walls, and a reduction in the thickness of the multi-layered biofilm growth can be seen. Furthermore, in the presence of a crude biosurfactant of *L. rhamnosus*, all bacterial strains failed to develop as clusters, as well as were unable to maintain their typical morphology. This is due to the compromised cell walls. One more essential studied aspect was EPS, which is produced by the bacteria and is vital for not only maintaining the structural integrity, but also substantially contributing to adhesion to various surfaces and microcolony development, leading to the formation of biofilms [61]. Our study displayed remarkable results in inhibiting the EPSs of all the test strains by the crude biosurfactant of *L. rhamnosus.* An EPS-rich matrix is very crucial for maintaining the physical stability and attachment of biofilms [62]. Therefore, targeting the biochemical constitution of EPS ultimately destabilizes the biofilm matrix and its complexity, which further eases the drug access directly into the biofilms [63].

The antibiofilm and anti-adhesion ability of the LAB-derived biosurfactants has been reported towards various microbial pathogens [23,27,59,64,65,66,67]. Therefore, studying the antibiofilm and anti-adhesion potential of LAB-derived biosurfactants might be considered as important to understand the mechanism behind it and identifying the potent chemical constituents in the crude biosurfactant responsible for combating the colonization of pathogenic microbes on different type of surfaces [65,68]. Sambanthamoorthy et al. (2014) extracted cell-bound biosurfactants from the *L. rhamnosus* 7469 strain and reported its antimicrobial, anti-adhesive and antibiofilm activities against *A. baumannii*, *E. coli* and *S. aureus* [69]. The biosurfactant derived from *L. helveticus* MRTL91 had lower anti-adhesive activity against *S. typhi*, *E. coli*, *P. aeruginosa*, *S. flexneri* and *C. albicans* at the same concentration. Falagas and Makris (2009) reported the anti-adhesion potential of biosurfactants isolated from the probiotic microorganism against the colonization of pathogens onto medical equipment/implants to control the nosocomial infections in hospitals [25]. Rodrigues et al. (2006) reported the application of an *S. thermophilus*-derived biosurfactant to inhibit the colonization of microbial pathogens on silicone rubber [27]. Gudina et al. (2010) reported the anti-adhesive ability of LAB-derived biosurfactants, and among those, the highest anti-adhesive activity was found against *S. aureus*, *S. epidermidis* and *S. agalactiae* at a concentration of 25 mg/mL [70]. The antibiofilm potentiality of the biosurfactants derived from two LAB strains, *L. paracasei* and *L. paracasei* ssp. *paracasei* A20, has been also reported against yeast and several human bacterial pathogens, and about 75% inhibition was found. Fracchia et al. (2010) also reported an 85% inhibition of biofilm at a biosurfactant concentration of 312.5 µg/mL derived from *Lactobacillus* spp. [71]. The complete inhibition of biofilm was found for a silicone tube at a concentration of 25 mg/mL of a biosurfactant derived from *L. helveticus* MRTL 91 [27]. More than 50% of the biofilm inhibition of different bacterial pathogens, such as *E. coli, S. aurues*, *C. albicans*, *E. faecalis* and *S. epidermidis*, has been reported by biosurfactants derived from *L. acidophilus*. The biosurfactants derived from *L. fermentum* B54 also showed anti-adhesive activity against uropathogenic microorganisms [54,62]. A few more strains of LAB, which are able to produce biosurfactants, are also reported to decrease the biofilm formation of several pathogens [27,64,72]. Biosurfactants derived from *L. lactis* 53 also inhibit the growth of *R. cariosa* and *C. tropicalis* on silicone tubing.

Moreover, bioactive compounds known to have antibacterial and antibiofilm potential present within the crude biosurfactants of *L. rhamnosus* were identified via GC–MS analysis (Table 3). From the identified bioactive compounds, Pyrrolo[1,2-a]pyrazine-1,4-dione,hexahydro-3-(phenylmethyl)-Cyclo(D-phenylalanyl-L-prolyl) is well-known for its potent inhibitory effect on multidrug-resistant *S. aureus* and other pathogenic bacteria [73]. Mannofuranoside and its derivatives are also reported as strong antimicrobial agents, specifically against pathogenic fungi and several pathogenic Gram-positive and Gram-negative bacteria [74]. 1-Heneicosanol is known for its antibacterial and antifungal activities [75] as well as for its anti-tuberculosis activity [76]. Identified 9-Octadecene, (E)-, 1-Heptadecene, *n*-Hexadecanoic acid are non-polar components, which are reported for their efficient antimicrobial activity against different pathogenic bacteria, such as *E. coli, P. aeruginosa, B. subtilis, S. aueus,* *E. faecalis*, *S. pneumoniae*, *P. mirabilis* and a fungus/yeast (*C. albicans*) [77,78,79]. Cyclo(L-prolyl-L-valine) is a cyclic dipeptide known as Diketopiperazines. It has been reported to be produced from different microbial species and is known to possess antitumor, antiviral, antifungal, antibacterial, anti-prion and anti-hyperglycemic activities [80].

## 4. Materials and Methods

### 4.1. Isolation and Screening of Lactic Acid Bacteria

Isolation of probiotic LAB was carried out from human breast milk. The sample was transferred in a flask consisting de Man, Rogosa and Sharpe (MRS) broth (Hi-Media^®^, Mumbai, India) (100 mL) as enrichment media and incubated at 37 °C for 24 h. After the incubation period, 100 μL of the enriched sample was spread on MRS agar plates with incubation in anaerobic conditions for 48 h at 37 °C. Subsequently, purified bacterial colonies were sub-cultured. The purified bacterial colonies were maintained on an MRS agar medium for immediate use and stored at −20 °C in 20% glycerol for future use.

### 4.2. Identification of Lactic Acid Bacteria

Identification of isolated lactic acid bacterial strain was carried out via the 16S rRNA gene sequencing method. Genomic DNA was extracted using bacterial genomic DNA kit (GenElute^TM^, Sigma-Aldrich, Bangalore, India). Quantification was carried out according to the method described by Sambrook et al. (1982) [81]. Optical density (OD) was measured (UV-1800, Shimadzu Spectrophotometer, Tokyo, Japan) at 260 and 290 nm. Further, 0.8% agarose gel was used to confirm the purity of the extracted genomic DNA by electrophoresis. 16S rRNA gene amplification was carried out by using a pair of universal primers 27f (5′AGAGTTTGATCCTGGCTCAG3′) and 1492r (5′CGGTTACCTTGTTACGACTT3′). A final volume of 20 µL was used for the PCR amplification containing 10 pmol of each primer, 1× ReadyMix™ Taq PCR reaction mix (Sigma-Aldrich^®^, Bangalore, India) and ~50 ng of template DNA, and to make up the total volume, nuclease-free water was added. The PCR cycling conditions were: 95 °C for 4 min, 35 cycles of 95 °C for the 30s, 54 °C for 30s, and 72 °C for 1 min, followed by a final extension step at 72 °C for 5 min with a hold at −4°C for ∞ time in a Thermal cycler (Applied Biosystems Veriti^®^, Lenexa, KS, USA). 1% agarose gel was used to detect the amplified PCR products by electrophoresis. Staining was performed by ethidium bromide (EtBr), followed by visualization under UV light. Further purification of the amplified PCR product was done using the GenElute™ PCR Clean-up kit (Sigma-Aldrich^®^, Bangalore, India) and the purified PCR product was sequenced. Finally, the Basic Local Alignment Search Tool (BLAST) on NCBI was used to carry out the sequence match analysis and sequences were later submitted to GenBank.

### 4.3. Biosurfactant Assays

Firstly, the isolated LAB strains were screened for its qualitative biosurfactant production ability via the following different assays.

#### 4.3.1. Emulsification Assay

The potentiality of the biosurfactants to emulsify n-hexadecane was carried out via an emulsification test [82]. Equal volumes of n-hexadecane and a cell-free biosurfactant solution were mixed by vortexing for 2 min and left to stand for 24 h. Calculation of an emulsification index (% EI24) was done by using the following equation:

(1) $$\%\mathrm{EI}24=\frac{\mathrm{Height}\;\mathrm{of}\;\mathrm{formed}\;\mathrm{emulsion}}{\mathrm{Total}\;\mathrm{height}\;\mathrm{of}\;\mathrm{the}\;\mathrm{solution}}\times 100$$

#### 4.3.2. Drop-Collapse Assay

The method described by Plaza et al. (2006) was followed to perform the drop-collapse test [83]. Firstly, a drop (35 μL) of cell-free biosurfactant solution was put onto parafilm for observation of drop spreading on the parafilm surface after 15 min. The collapsed drop was scored as a positive result, which indicated the presence of biosurfactants.

#### 4.3.3. Oil-Spreading Assay

The method described by Joe et al. (2019) was followed to perform the oil-spreading assay [84]. Firstly, distilled water (50 mL) was added to the Petri plates, followed by vegetable oil (100 μL) on the surface of the water. Later, on to the oil surface, 10 μL of the cell-free biosurfactant solution was put. After 30 s, the surface of the oil was visualized for the development of a clear zone.

#### 4.3.4. Blue Agar Plate (BAP) Assay

A BAP assay was carried out for the detection of biosurfactants on a minimal agar medium consisting of cetyl trimethyl ammonium bromide (C-TAB, 0.4 mg/mL), glucose (2%) and methylene blue dye (0.2 mg/mL). Wells of 6 mm in size were made into the plates with the help of a sterile cork borer and 20 µL of a cell-free biosurfactant solution was added into the wells. Plates were left in a refrigerator for 30 min for the diffusion of the spent broth and kept for incubation for 24 h at 37 °C. After the incubation period, the presence of a dark blue color halo around the wells were observed in the plates.

### 4.4. Surface Tension Measurements

The surface tension was measured with a tensiometer (K11, Krus, Germany). Pure water was used as a standard before reading (72.50 mN/m).

### 4.5. Control Solutions

In the screening assays, phosphate-buffered saline (PBS) was used as a negative control and sodium dodecyl sulphate (SDS) was used as a positive control.

### 4.6. Production of Biosurfactant and Extraction

The production of biosurfactants by the isolated LAB strain was carried out via growing them in MRS-Lac broth (cultivation medium for LAB, where glucose is substituted by lactose). For the production of the crude biosurfactant, an overnight grown culture of *L. rhamnosus* (1%) was inoculated in MRS-Lac (1000 mL) medium and incubated at 37 °C for 48 h without shaking. After the incubation period, a culture medium was placed for centrifugation (10,000 rpm, 10 min, 10 °C) to harvest the cells. Next, demineralized water was used to wash the cells twice and further re-suspended in 100 mL of phosphate-buffered saline (PBS) (pH = 7.0). This solution was stirred gently at room temperature for 4 h to release the cell-associated biosurfactant. After 4 h, centrifugation was carried out to remove the bacteria and the supernatant was collected by filtering with 0.22 µm filter. In the end, the filtered and sterilized supernatant was lyophilized, stored at −40 °C and further resuspended in deionized water at 100 mg/mL. This solution of the crude biosurfactant was then used for the biosurfactant assay and biofilm eradication assay.

### 4.7. Assessment of Biomass and Biosurfactant Concentration

Growth of the bacteria was investigated by taking the OD of the broth at 600 nm. For the determination of biomass, a regular method for the measurement of cell dry weight was employed. A total 10 mL of the bacterial sample was transferred into pre-weighed tubes and centrifuged at 10,000 rpm for 10 min. Lastly, the cell pellet was collected and oven-dried for 24 h at 100 °C and the dry weight was estimated eventually. The biosurfactant concentration was determined according to the procedure described above (Section 4.6) and the concentration of the produced biosurfactant was represented in mg/mL.

### 4.8. Assessment of Physical Properties of Biosurfactant

To investigate the %EI24 of the produced biosurfactant by *L. rhamnosus*, an equal volume of n-hexadecane and biosurfactant solution was mixed for 2 min vortexing and left to stand for 24 h. Then, calculation of an emulsification index (%EI24) was carried out as described above. Beside from n-hexadecane, the %EI24 of the produced biosurfactant was also investigated against various substrates such as gasoline, diesel, kerosene, toluene, olive oil and sunflower oil. Further, using a surface tensiometer, values of CMC and ST reduction were evaluated. CMC is defined as the abrupt discontinuity in the surface tension plot versus the plot of biosurfactant concentration. 

### 4.9. Assessment of Antibacterial Activity

All pathogenic bacterial strains, *B. subtilis* (MTCC 121), *P. aeruginosa* (MTCC 741), *S. aureus* (MTCC 96) and *E. coli* (MTCC 9537), were obtained from the Microbial Type Culture Collection (MTCC), India, and maintained on Muller-Hinton Agar (MHA). The antibacterial activity of the *L. rhamnosus* crude biosurfactant and standard SDS was carried out via the agar cup/well diffusion method. Firstly, bacterial cultures were grown overnight at 37 °C in a fresh medium and a total of 0.5 Mc Farland standard 10^8^ colony-forming units/mL (CFU/mL) was matched by culture turbidity adjustment using a sterile saline solution. The bacterial suspension was evenly spread all over the plates and wells were made with a sterile cork borer. A total of 60 µL of the biosurfactant solution (100 mg/mL) and SDS (1% *v*/*v*) solution was then inoculated into each respective well and plates were incubated at 37 °C for 24 h. Antibacterial activity was noted in the form of zone of inhibition. Chloramphenicol and sterile water were used as the positive and negative control, respectively.

### 4.10. Minimum Inhibitory Concentration (MIC) and Minimum Bactericidal Concentration (MBC) Determination

MIC determination of the *L. rhamnosus* crude biosurfactant and standard SDS was carried out in microtiter plates (96-well) against the tested bacterial strains, as reported previously [85]. The inoculums were prepared from 12 h Muller-Hinton broth (MHB) culture. The *L. rhamnosus* crude biosurfactant was diluted to two-fold ranging from 100 to 1.56 mg/mL in MHB in a 96-well plate (100 µL each well). Similarly, standard SDS was also diluted (1 to 0.2%). A diluted culture of each test bacteria was added to the respective well to control the final concentration of 10^8^ CFU/mL, followed by being incubated for 24 h at 37 °C. The MIC was then recorded as the lowest concentration at which absolute inhibition of observable growth occurred. Wells with media were used as a negative control, whereas wells without biosurfactant in media but only inoculated bacteria were used as a positive control. Similar to the MIC assay, MBC determination of the *L. rhamnosus* crude biosurfactant was also carried out by spreading 5 µL of sample from the wells, which exhibited no evident growth on MHA plates, and kept for incubation for 24 h at 37 °C. The MBC was recorded as the lowest concentration at which 99% of the inoculum was killed; i.e., three or fewer colonies [86].

### 4.11. Preparation of Biofilm 

The crystal violet (CV) method was followed for determining the biofilm-forming ability of the tested strains using 96-well polystyrene plates [87]. Briefly, a log-phase culture of each test strain with MHB (200 µL) at an initial turbidity of 0.05 at 600 nm was incubated for 24 h at 37 °C without shaking. After the incubation period, planktonic cells were removed by washing thrice with PBS and air-dried. A 0.1% CV was then used for staining the wells and kept for 20 min. By dissolving in 95% ethanol, the excess amount of dye was taken out and absorbance was measured at 570 nm.

### 4.12. Antibiofilm Assays

#### 4.12.1. Effect of the *L. rhamnosus* Crude Biosurfactant on the Established Biofilms 

The method described by Lemos et al. (2018) was followed to assess the efficacy of the *L. rhamnosus* crude biosurfactant and standard SDS on established biofilms. 96-well microtiter plates were used to form the biofilms by test strains containing 1% glucose, MHB and cells (10^7^ cells/mL) [60]. Plates were incubated for 24 h at 37 °C. After incubation, planktonic cells were delicately removed from the wells with further washing of the wells with saline thrice. After washing, the *L. rhamnosus* crude biosurfactant and standard SDS (MIC) (200 µL) were added to the respective wells and plates were kept for another day (24 h) of incubation at 37 °C. Absorbance (492 nm) was measured at 0 and after 24 h. The MHB medium without any surfactant and with individual test strain was used as the biofilm growth control and the biofilm eradication percentage was calculated as

[(OD (control) − OD (test)/OD (control)] × 100(2)

#### 4.12.2. Effect of the *L. rhamnosus* Crude Biosurfactant on Adherence of Biofilms 

The method described by Plyuta et al. (2013) was followed to determine the effect of the *L. rhamnosus* crude biosurfactant and standard SDS on the adherence of biofilms formation [88]. The bacterial cell culture (100 µL) of each tested strain (10^8^ CFU/mL) and *L. rhamnosus* crude biosurfactant and standard SDS (MIC) collectively in the respective 96-well microtiter plates were incubated for 24 h at 37°C. After incubation, planktonic cells were delicately removed from the wells with further washing of the wells with PBS (200 µL). After washing, the adhered cells were stained with 0.1% CV for a 30 min incubation at 37 °C to visualize the developed biofilms by test strains. Excess CV dye was washed off with PBS and plates were finally fixed using 95% ethanol (200 µL) and incubation for 15 min at 37 °C. Absorbance at 590 nm was then measured. Inhibition percentage was calculated as

[(OD (control) − OD (test)/OD control)] × 100(3)

### 4.13. Microscopic Assessment

#### 4.13.1. Determining and Visualization of Antibiofilm Activity by Light Microscopy

The method described by Musthafa et al. (2010) was followed to investigate the biofilms formed by the test strains using LM with slight modifications [89]. 24-well microtiter plates consisting of 1 × 1 cm size cover slips were inoculated with 500 µL of the test cultures (10^8^ CFU/mL). In the same well, 500 µL of the *L. rhamnosus* crude biosurfactant (final concentration = MIC) was added as the treatment. For the positive control, the same volume of chloramphenicol was used with the test strains. For the negative control, the same volume of sterile water was used with the tested strains. After the incubation period of 24 h at 37 °C, glass cover slips with the formed biofilms were gently removed and washed with PBS. Staining was performed with 0.1% CV as described above, followed by washing and air-drying for 5 min. Cover slips stained with CV were then observed under LM with 40x magnification (Axioscope A1, ZEISS, Oberkochen, Germany).

#### 4.13.2. Determination and Visualization of Antibiofilm Activity by Scanning Electron Microscopy

All the tested strains’ biofilms were also analyzed by SEM (with and without the *L. rhamnosus* crude biosurfactant as well as the respective controls as described above). First, the biofilms were fixed on glass coverslips using 2.5% glutaraldehyde at 37 °C for 30 min. After fixing, cover slips were washed thrice with PBS and then dehydrated through a graded series of ethanol solution (30%, 50%, 70%, 90% and 100%) at 15 min intervals. Samples were then freeze dried after reinstation of ethanol with isoamyl acetate. Finally, using E-1010 ion sputter (Hitachi^®^, Tokyo, Japan), cover slips were coated with gold and observed under SEM (S-34002N SEM, Hitachi^®^, Japan).

### 4.14. Bacterial Metabolic Activity in the Biofilm Assays

To determine the viability of the bacterial cells within the biofilms a colorimetric XTT reduction test was performed [66,90,91]. Briefly, a log phase culture of each test strain collectively in MHB (200 µL) with and without the *L. rhamnosus* crude biosurfactant at an initial turbidity of 0.1 at 600 nm was incubated for 24 h at 37 °C without shaking. After the incubation period, planktonic cells were removed by washing thrice with distilled water, followed by sterile PBS (100 µL). After washing, the XTT-menadione (100 µL) solution (freshly prepared) was added into the wells. Plates were then incubated in the dark at 37 °C for 5 h. Following the incubation, a colored supernatant (100 µL) was transferred from each well to a new 96-well microtiter plate. A microplate reader was then used to record the absorbance at 480 nm. The survival percentage of the bacterial population was determined as follows:

[(OD (biosurfactant treated sample) − OD (negative control)/OD of untreated control)] × 100(4)

### 4.15. Bacterial Cell Damage Assay

To determine the damage to bacterial cells within the biofilms, an LDH assay was carried out. Briefly, the log phase culture of each test strain (100 µL) with MHB (100 µL) was added into 96-well microtiter plates and incubated for 24 h at 37 °C under static condition. After the incubation period, planktonic cells were discarded by washing thrice with sterile PBS (100 µL). The *L. rhamnosus* crude biosurfactant (MIC) (100 µL) was then added and the plates were kept for further incubation at 37 °C for 24 h under static conditions. After incubation, the LDH activity was then determined by collecting the supernatant using an LDH assay kit (Sigma-Aldrich^®^, Bangalore, India) at 480 nm. The bacterial culture and MHB was used as the negative control.

### 4.16. Determining the Production of Exopolysaccharide by Ruthenium Red Staining

To determine the activity of *L. rhamnosus* crude biosurfactant in diminishing the EPS matrix production of biofilm, a Ruthenium red staining assay was carried out [92]. The log-phase culture of each test strain (100 µL) and *L. rhamnosus* crude biosurfactant (MIC) were incubated for 24 h at 37 °C. After the incubation period, planktonic cells were discarded by washing thrice with sterile PBS (200 µL). Formed and structured biofilms by the adherent cells were then stained with 0.01% Ruthenium red (Sigma-Aldrich^®^, Bangalore, India) (200 µL). One well without biofilm and with Ruthenium red served as the blanks. Plates were then kept for incubation at 37°C for 1 h. Liquid holding the residual stain was resettled in a new microtiter plate and the absorbance was read at 450 nm. The quantity of the dye fixed to the biofilm matrix was measured as follows:

Abs_BF_ = Abs_B_ – Abs_S_ (5)

where Abs_B_ = absorbance of the blanks, and Abs_S_ = absorbance of the residual stain collected from the sample wells

### 4.17. Gas Chromatography–Mass Spectophotometry (GC–MS) Analysis

The identification of the structural analog of the crude biosurfactant was carried out using a GC apparatus (GC-2030, Nexis, Shimadzu^®^, Kyoto, Japan) equipped with QP2020 NX-MS. A total of 10 µL of the sample was injected into the system and helium was used as a carrier gas. A flow rate of 1 mL/min was used and the run time was 20 min. The oven temperature was kept between 60 °C and 260 °C. The MS of the detected compounds was compared with the National Institute of Standards and Technology (NIST) database.

## 5. Conclusions

Collectively, this study revealed that the *L. rhamnosus*-derived biosurfactant has significant antibacterial potential against diverse Gram-positive and Gram-negative pathogenic bacteria. The crude biosurfactant derived from *L. rhamnosus* also displayed potent inhibition of biofilm and bacterial adhesion via influencing the viability and integrity of the bacterial cells within the biofilms, as well as by hampering the production of EPS. These findings indicate that the *L. rhamnosus* crude biosurfactant can potentially be useful or can become a potent antibacterial and antibiofilm compound, as an alternative to antibiotics or other chemically synthesized toxic agents. Hence, we recommend more investigations to be conducted to have a better understanding about the broad action of crude biosurfactants, before efforts are made to develop its pharmaceutical/industrial applications.

## Figures and Tables

**Table 1:** Qualitative and quantitative results of the different screening assays for the production of biosurfactants (values are the mean ± SD (*n* = 3)).

Strain	Colony Characteristics	Gram’s Reaction	Oil-Spreading Test	Drop-Collapse Test	BAP Test	%EI24 (n-Hexadecane)	ST (mN/m)
*L. rhamnosus* -MBP002	White, circular, shiny appearance	Gram-positive, rod shaped	Positive	Positive	Positive	32.37 ± 1.26	47.62 ± 1.47

**Table 2:** Antibacterial activity of the *L. rhamnosus* crude biosurfactant and standard SDS.

Bacterial Strain	*L. rhamnosus* Crude Biosurfactant (mg/mL)		SDS (%)	
	MIC	MBC	MIC	MBC
*B. subtilis*	12.5	25	0.2	0.4
*E. coli*	12.5	25	0.4	0.6
*P. aeruginosa*	25	50	0.6	0.8
*S. aureus*	50	100	0.8	1

**Table 3:** Major constituents of the crude biosurfactants of *L. rhamnosus* using GC–MS.

No.	RT	% Area	Compound Name	Class
1	1.914	47.87	Isopropyl alpha-D-mannopyranoside	Glycoside
2	2.422	4.98	2,5-Monomethylene-l-rhamnitol	Sugar Alcohol
3	13.233	0.92	9-Octadecene, (E)-	Fatty Acyl
4	15.753	1.13	1-Heptadecene	Fatty Acyl
5	17.589	1.51	Pyrrolo[1,2-a]pyrazine-1,4-dione,hexahydro-3-(phenylmethyl)- Cyclo (D-phenylalanyl-L-prolyl) (Cyclo(Phe-Pro))	Dipeptide
6	18.008	1.71	1-Heneicosanol	Fatty alcohol
7	18.249	4.86	Cyclo(L-prolyl-L-valine)	Diketopiperazine (Dipeptide)
8	18.473	1.10	7-Ethyl-4,6-heptadecandione	Fatty Acyl
9	19.426	4.15	*n*-Hexadecanoic acid	Surfactant

## Data Availability

All data generated or analyzed during this study are included in this article.
